# Linking brain stroke risk factors to human movement features for the development of preventive tools

**DOI:** 10.3389/fnagi.2014.00150

**Published:** 2014-07-08

**Authors:** Christian O'Reilly, Réjean Plamondon, Louise-Hélène Lebrun

**Affiliations:** ^1^Laboratoire Scribens, Département de Génie Électrique, École Polytechnique de MontréalMontréal, QC, Canada; ^2^Dream and Nightmare Laboratory, Center for Advanced Research in Sleep Medicine, Hôpital du Sacré-Coeur de MontréalMontréal, QC, Canada; ^3^Department of Psychiatry, University of MontrealMontréal, QC, Canada; ^4^Département de Neurologie Vasculaire, Centre Hospitalier de l'Université de Montréal, Hôpital Notre-DameMontréal, QC, Canada

**Keywords:** brain stroke, human movement science, lognormal models, prevention tools, kinematical analysis, stroke risk factors

## Abstract

This paper uses human movement analyses to assess the susceptibility of brain stroke, one of the most important causes of disability in elders. To that end, a computerized battery of nine neuromuscular tests has been designed and evaluated with a sample of 120 subjects with or without stoke risk factors. The kinematics of the movements produced was analyzed using a computational neuromuscular model and predictive characteristics were extracted. Logistic regression and linear discriminant analysis with leave-one-out cross-validation was used to infer the probability of presence of brain stroke risk factors. The clinical potential value of movement information for stroke prevention was assessed by computing area under the receiver operating characteristic curve (AUC) for the diagnostic of risk factors based on motion analysis. AUC mostly varying between 0.6 and 0.9 were obtained, depending on the neuromuscular test and the risk factor investigated (obesity, diabetes, hypertension, hypercholesterolemia, cigarette smoking, and cardiac disease). Our results support the feasibility of the proposed methodology and its potential application for the development of brain stroke prevention tools. Although further research is needed to improve this methodology and its outcome, results are promising and the proposed approach should be of great interest for many experimenters open to novel approaches in preventive medicine and in gerontology. It should also be valuable for engineers, psychologists, and researchers using human movements for the development of diagnostic and neuromuscular assessment tools.

## Introduction

A brain stroke, or cerebrovascular accident, is characterized by the sudden loss of brain functions caused by an interruption of the blood supply to the brain or by the rupture of blood vessels in the brain. Its prevalence increases markedly with aging. Each years, 50,000 Canadians and 795,000 Americans are victims of this disease. In Canada and United-States, it is the third cause of mortality, causing direct and indirect costs of Can$ 3.6 billion (evaluated in 2000) and US$ 68.9 billion (evaluated in 2009) (Lloyd-Jones et al., 2009; Fondation des maladies du coeur, 2012). For surviving patients, the consequences can be various. Among subjects of 65 years old or more of the Framingham Study cohort, 6 month post-stroke, 50% were suffering from hemiparesis, 30% were unable to walk without assistance, 26% needed assistance for their daily activities and 26% were institutionalized. Also, aphasia, depression, incontinence, sensitive deficits, social integration difficulties, and hemianopsis were frequent symptoms (between 15 and 40%) (Kelly-Hayes et al., 2003).

Although brain strokes are unexpected outcomes which strike suddenly, the scientific literature reports for example, that some events are known to happen significantly more often before brain strokes such as transient ischemic attack (Hankey, 1996), silent brain infarction (Kobayashi et al., 1997; Bokura et al., 2006), and pre-stroke dementia (Klimkowicz et al., 2004). These phenomena may be indicative of a particular pre-stroke state of the cerebrovascular system. Moreover we have shown recently that brain stroke risk factors can be associated with the deterioration of many cognitive and psychomotor characteristics (O'Reilly and Plamondon, 2011). It is in this context that we investigate in this paper if the state of the neuromuscular system of a person might be indicative of an incoming brain stroke. If this is the case, covert and overt responses to psychomotor tests could be expected to correlate with the brain stroke susceptibility and possibly be used for prevention.

In this context, it might be interesting to look at the motor control for pre-stroke markers. Although somewhat unconventional, this kind of approach follows a fruitful line of investigations using pattern recognition analyses of fine motor control for biomedical applications. This has been the case for example for studying Parkinson disease (Van Gemmert et al., 2003), Alzheimer disease (Werner et al., 2006), and schizophrenia (Caligiuri et al., 2009), as well as for designing neuropsychological tests (Fairhurst et al., 2008; O'Reilly and Plamondon, 2010a) for the detection of various health problems or for the recovery from injuries (Yancosek and Mullineaux, 2011).

The present paper reports on a feasibility study verifying the potential of this approach by analyzing the possibility of assessing the brain stroke susceptibility through motion analysis. In this line of thoughts, we have gathered a transversal database of 120 subjects who have performed a variety of neuromuscular tests. In this paper, we present a summary of the outcome of our research program in terms of area under the receiver operating characteristic curve (AUC) for the classification of brain stroke risk factors as a demonstration of the level of clinical potential of human movement information for the development of brain stroke prevention tools.

## Methods

### Sample

One hundred and twenty volunteers recruited within the École Polytechnique community and from the patients of a rehabilitation hospital (Hôpital De Réadaptation Villa Medica) participated in the experiment. They were taken from a wide age range (25–85 years old) and from both genders (68 women, 52 men).

Eight participants had a stroke in the past and 63 had some of the following health risk factors (abbreviation; number of subjects affected): diabetes mellitus (DM; 15), obesity (OB; 10), hypertension (HT; 40), hypercholesterolemia (HC; 28), cardiac disease (CD; 24), and cigarette smoking (CS; 13). From these 63 participants, 25 had only one risk factor, 18 had two, 12 had three, seven had four, and one had five. The other 57 were free of these risk factors and were considered as healthy. Risk factors were evaluated from a medical form processed by a neurologist (Dr. L.-H. Lebrun) for the subjects from École Polytechnique whereas, for the hospital patients, this information was collected by the same neurologist from the medical records of the patients. The experimenters were kept blind in regard of the presence of stroke risk factors in the subjects.

**Figure 1:**
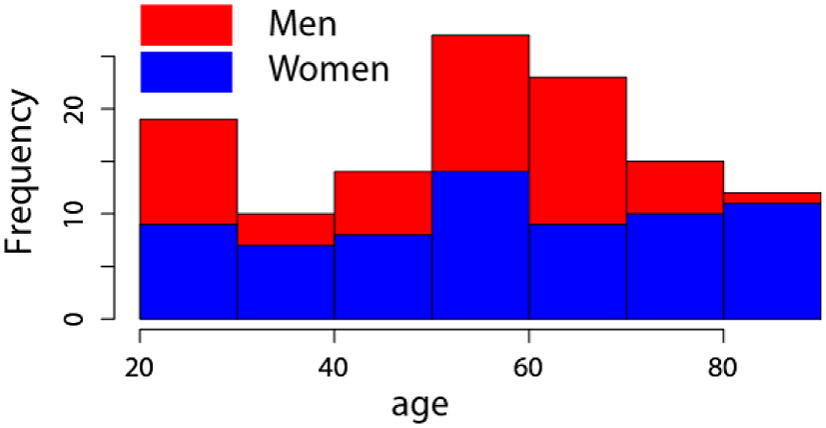
**Distribution of age, for both gender, in our sample**.

From our sample, 112 participants reported themselves as right-handed, seven as left-handed and one as ambidextrous. The participants performed the experiment with their dominant hand (the ambidextrous one used his right hand) and the left-handed were given reversed guiding sheets to mitigate the effect of the hand used in the experiment. Figure 1 shows the distribution of the age for both gender. A more complete description of this sample can be found elsewhere (O'Reilly and Plamondon, 2011; O'Reilly, 2012).

Every participant in this experiment received a brochure explaining the experiment and gave an informed written consent. The experimental protocol was approved by the ethics board of the École Polytechnique de Montréal and of the Hôpital de Réadaptation Villa Medica.

### Material

An in-house system was built to emit real-time (1 ms accuracy) audio and visual stimuli to the subjects and to accurately track the end-effector of the movement performed as response. Both features are essential for modern computerized modeling of human movements performed in responses to stimuli. Movements were captured using a Wacom Intuos2 digitizing tablet, which can record 2D Cartesian coordinates of a pen tip (either a stylus or an inking pen, depending on whether the feedback provided by the inked trace is wanted) at 200 Hz and with a 100 lines per millimeter spatial resolution. Guiding sheets were used to indicate to the subjects the starting positions and the targets to hit. These sheets were placed under the transparent plastic fold of the tablet to reduce friction. The patterns of these guiding sheets were reversed for left-handed subjects such that, for a given task, the same muscles are involved for both right and left-handed participants. A stimulator—an apparatus allowing to emit auditory (1 kHz beep of a 500 ms duration) and visual (various patterns displayed on a matrix of 8 × 10 light-emitting diodes [LED] with three possible colors: green, red, yellow) stimuli—linked through the serial port of the data acquisition computer was used for the interaction with the subject. Synchronization between the Wacom tablet and the stimulator was performed by an in-house software name Sign@medic, which also managed the experiment workflow and the data recording.

### Neuromuscular testing

Since no neuromuscular test was readily available to investigate our research hypotheses using modern movement modeling tools, it was necessary to synthesize a new battery of tests from information taken in the psychophysical literature. Thus, different tests have been derived to assess, through the analysis of movement kinematics, the performances of the subject with respect to various cerebral functions. This protocol was designed, tested and optimized, over 2 months, with a few subjects from both groups (not included in the present group of participants) until it was established that the instructions were clearly understood by the participants. The order of the tasks and the number of repetitions were also fixed taking into consideration that the participants, particularly the aged ones, should not be tired at the end of the data collection.

The next subsections present—in the same sequential order used to test the subjects—the corresponding literature and the description of the nine neuromuscular tests constituting this battery.

#### Task #1: initial signatures

Handwriting movements have been extensively studied along the years (e.g., Simner et al., 1996; Caligiuri and Mohammed, 2012 and the proceedings of the International Graphonomics Society biannual conferences) and their usefulness in medical diagnostic has been demonstrated (Schroter et al., 2003; Van Gemmert et al., 2003; Caligiuri et al., 2010). The first test of our battery takes advantage of this gathered knowledge by proposing a task requiring the production of a special kind of handwriting movements: the handwritten signatures. The signing process involves highly complex fine motor control to generate a mostly ballistic and overlearned movement.

**Figure 2:**
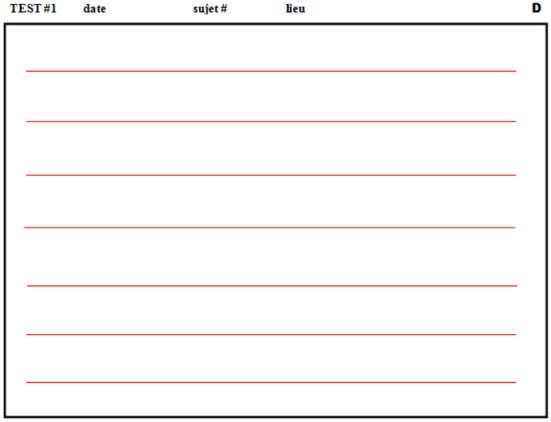
**Guiding sheet used to collect handwritten signatures**.

In this test, four samples of handwritten signature have to be performed using an inking Wacom pen. The subject is allowed to signal if he/she feels that a produced written signature is not representative of his/her usual signature. In this case, the trial is rejected and a replacement specimen is collected. Figure 2 shows the guiding sheets on which the subject signs.

Between each signature, the subject must remove the pen from the tablet and wait for the system to be ready for recording, indicated by the stimulator by a blinking red screen. In this task, no other stimulus is emitted to indicate when the movement must start, leaving the subject free to sign repetitively at his/her own pace.

A signature verification system (Sign@matic) (Plamondon et al., 1992; Plamondon, 1994) is operated by Signa@medic. The three first signatures collected are used to register the subject to the system and the fourth one is used to verify if the subject can be recognized by comparing this signature with the first three references. If the system fails to recognize the fourth signature as belonging to the participant, it is probable that the signature set has a large variability and that the subject might not be in optimal physical conditions to perform the experiment. A fifth signature is then requested to check this hypothesis and allow a better characterization of the initial neuromuscular conditions.

#### Task #2: fast pen stroke/simple reaction to a visual stimulus

This test involve the visual areas and is based on the well-known simple reaction test (SRT) (Luce, 1986). It examines the capacity of the subject to react as quickly as possible to a visual stimulus. It is also based on the various forms of reaching tests (e.g., Levin, 1996; Lum et al., 1999; Minegishi and Takahashi, 2001; Prablanc et al., 2003; Wagner et al., 2008) since, contrarily to usual implementations of reaction time tests, which often only request pressing a button, we are interested by the kinematic of the whole movement of the upper-limb, from the starting point to the targeted zone.

**Figure 3:**
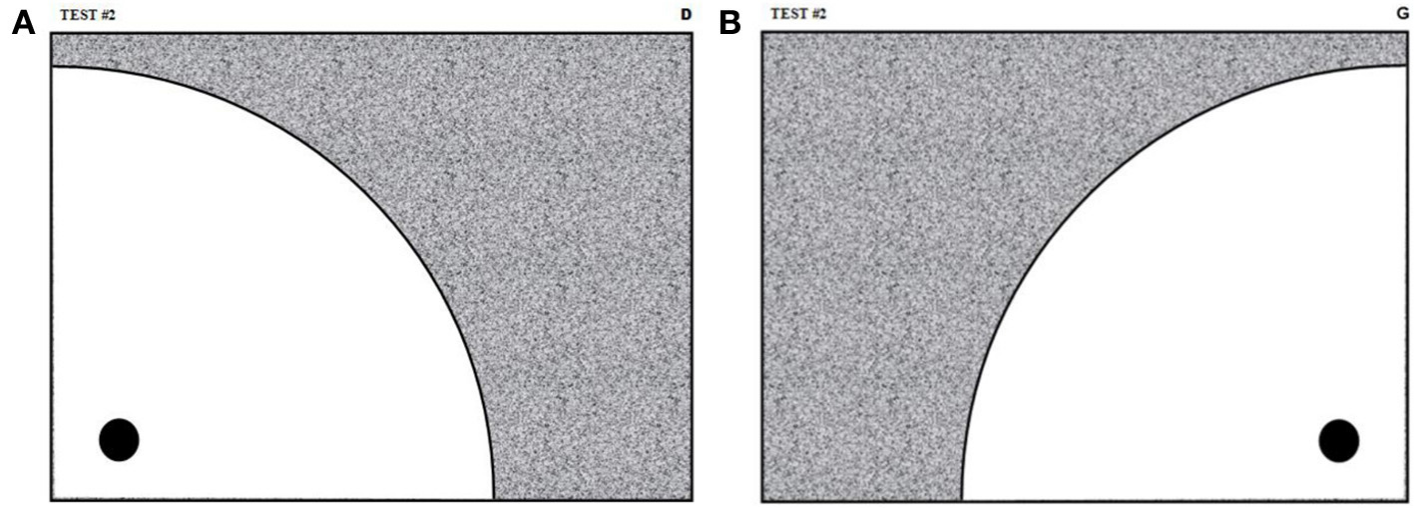
**Guiding sheets for the fast pen stroke test with simple stimulus**. The black circles and the gray areas indicate respectively the starting and the target zones. This sheet in **(A)** is used for right-handed subjects. Left-handed subjects are given identical guiding sheets except that they are mirror reflected with respect to a vertical symmetry axis, as shown in **(B)**.

In this test, neither the precision nor the direction is important, only the speed of execution of the movement is. The subject performs the test using a Wacom stylus over the tablet's transparent plastic fold and following the guiding sheet shown in Figure 3. In this figure, the black circle represents the starting point and the gray area is the target zone where the subject must stop his/her movement. The dimensions of this sheet are such that a valid movement (i.e., beginning in the starting zone and ending in the target zone) must have at least 130 mm of amplitude.

After the experimenter has shown to the subject an example of how the task must be done, the subject can try the experimental protocol as long as necessary to feel comfortable with the test and the equipment. Movements recorded during this learning period are automatically labeled as “in learning” and are discarded from the dataset used for statistical analysis.

When the participant signals that he/she is ready, the recording of at least 15 valid trials starts. Each trial follows this procedure:

1. The LED screen blinks from red to black to indicate that the system is ready for acquisition. The subject can position the tip of the stylus on the starting point.
2. At the moment the stylus hits the digitizer, the stimulator stops blinking and a random delay is started. This delay is exponentially distributed such that, regardless of the duration the subject has waited for the stimulus, the probability that it will be emitted during the next millisecond is always the same (Luce, 1986). The parameters of this flat hazard distribution have been chosen such that the delay is between 0 and 10 s.
3. As soon as the delay expires, the LED screen becomes green, signaling the subject to reach for and stop in the target zone as fast as possible.
4. After the pen tip has been immobilized in the target zone, the subject must lift the pen away from the tablet. When the stylus exits the active zone of the tablet^1^, the stimulator starts sending a red blinking signal and the procedure can be repeated for the required number of times.

When a subject starts moving before the stimulus emission, the stimulator shows a yellow blinking “X” and emits a beep informing the subject that an anticipated start has been made. These data are automatically labeled as invalid.

#### Task #3: fast pen stroke/choice reaction to a visual stimulus

This test evaluates the capacity of a subject to react quickly and to make a good choice at the same time, both characteristics being of equal importance. Compared to the SRT, the choice reaction test (CRT) involves decisional processes adding a layer of complexity and therefore increasing the average reaction time (Luce, 1986). As for the preceding test, this task also involves the visual areas.

**Figure 4:**
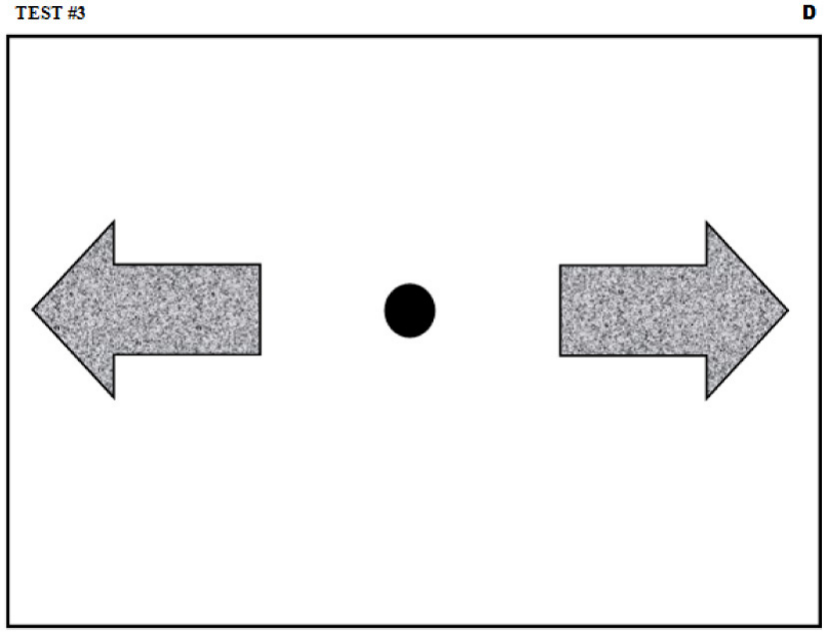
**Guiding sheet for the fast pen stroke on a choice visual stimulus**. The black circle and the gray areas indicate respectively the starting and the target zones.

The protocol for this test is identical to the preceding one, with two exceptions. First, 30 valid trials are recorded. Second, the guiding sheet used for the test is different and is shown in Figure 4. As can be seen, two target zones are placed on each side of the sheet (gray arrows) with the starting area (black circle) being at the center. Moreover, the stimulus used is changed for a green arrow indicating the required direction of the movement (i.e., if the stimulus arrow points to the right, the movement must be rightward). The direction of the stimulus (leftward vs. rightward arrow) is chosen randomly from trial to trial.

#### Task #4: fast pen stroke/simple reaction to an auditory stimulus

This test is identical to the test #2 except that it involves the auditory area. The trigger is a 1 kHz beep of 500 ms duration.

#### Task #5: speed/accuracy trade-off

Speed/accuracy trade-off (SAT) tests have been under a lot of investigation following the work of Fitts (Fitts, 1954; Fitts and Peterson, 1964)^2^. These seminal papers linked the average duration of the movement to the logarithm of a difficulty index being evaluated as the ratio between the amplitude of the movement and the width of the target. Speed/accuracy tradeoffs have been recognized as a good window into how the brain works when spatial and timing constraints are involved. Accordingly, this protocol has been adapted to study the behavior under a lot of different conditions such as in the presence of obstacles (Jax et al., 2007; Vaughan et al., 2010), with or without visual feedback (Wu et al., 2010), with different configurations of target geometry (Bohan et al., 2003), in presence of visual illusions (Van Donkelaar, 1999; Mendoza et al., 2005), with moving targets (Chiu et al., 2011), and so on.

**Figure 5:**
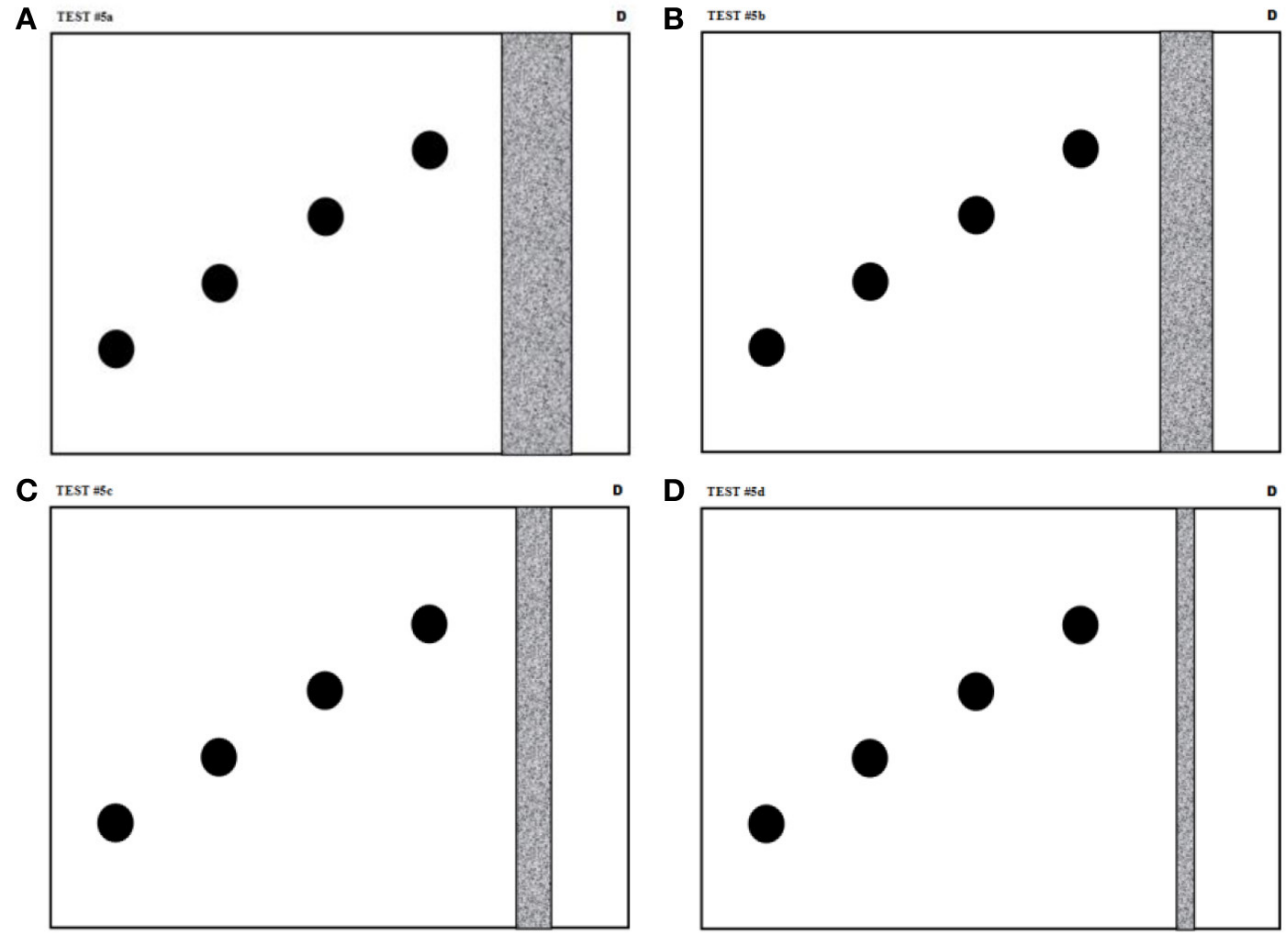
**Guiding sheets for the speed/accuracy trade-off task**. The black circles and the gray areas indicate respectively the starting and the target zones. The distance between the centers of the starting and the target zones are 45, 90, 135, and 180 mm and the width of the target zones are **(A)** 30 mm, **(B)** 22.5 mm, **(C)** 15 mm, and **(D)** 7.5 mm. These sheets are for right-handed subjects. Left-handed subjects are given guiding sheets that are identical except for being mirror reflected with respect to a vertical symmetry axis.

This task has been added to the battery to assess the ability of the participants to coordinate spatial and temporal properties of their movements under competing speed (temporal) and accuracy (spatial) requirements. A 4 × 4 factorial design with two repetitions have been used with the experimental factors being (1) the distance between the centers of the starting and the target zones (modalities: 45, 90, 135, and 180 mm) and (2) the width of the target zone (modalities: 30, 22.5, 15, and 7.5 mm). The guiding sheets used for this test are shown in Figure 5. On each of these, four starting points are indicated as black circles while the target zone is shown as a gray band.

The subject is allowed a learning period only on the first guiding sheet (Figure 5A). On every page, the subject begins the first trial in the starting circle closer to the target, and then moves to the next closer, and so on for the four starting positions. The participant then repeats these four movements once more. The identical procedure (except for the learning period) is followed with each guiding sheet, presented to the subject in decreasing order of target width.

For this task, the interaction with the stimulator is as described for the test #2 except that the auditory signal is used.

#### Task #6: fast pen stroke sequence

**Figure 6:**
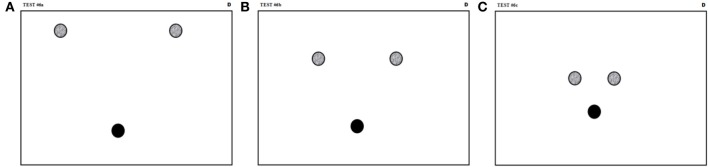
**Guiding sheets for the fast pen stroke sequence test**. The black circles indicate the starting and the gray circles specify the target zones that have to be crossed prior to coming back to the black dots. The targets are 15 mm in diameters and are positioned at the apexes of equilateral triangles with vertexes of **(A)** 135 mm, **(B)** 90 mm, and **(C)** 45 mm long.

This test evaluates the capacity of the subject to produce drawings requiring a sequence of fast pen strokes with both speed and accuracy. It allows studying the subject ability to coordinate motor command sequences. Guiding sheets used for this test are shown in Figure 6. In this test, the subjects must perform movements with triangular trajectories by sequencing three fast target reaching motions. The subject must start from the black circle, pass through the two intermediate targets (gray zones) and then stop the pen tip in the starting zone. As the movement must be performed as quickly as possible, the participant must not halt at intermediate targets.

The guiding sheets are presented in decreasing order of triangle size. Before starting valid recordings, the subject is allowed to practice as long as wanted, but only on the first sheet. On each sheet, the test must be performed (i.e., a triangular movement must be made) two times in clockwise directions and then two times in counter clockwise direction. Thus, 12 movements per subject are recorded in a factorial design 2 × 3 × 2 (rotation directions × triangle sizes × repetitions). The movement is initiated following an auditory stimulus.

#### Task #7: oscillations at maximal speed

**Figure 7:**
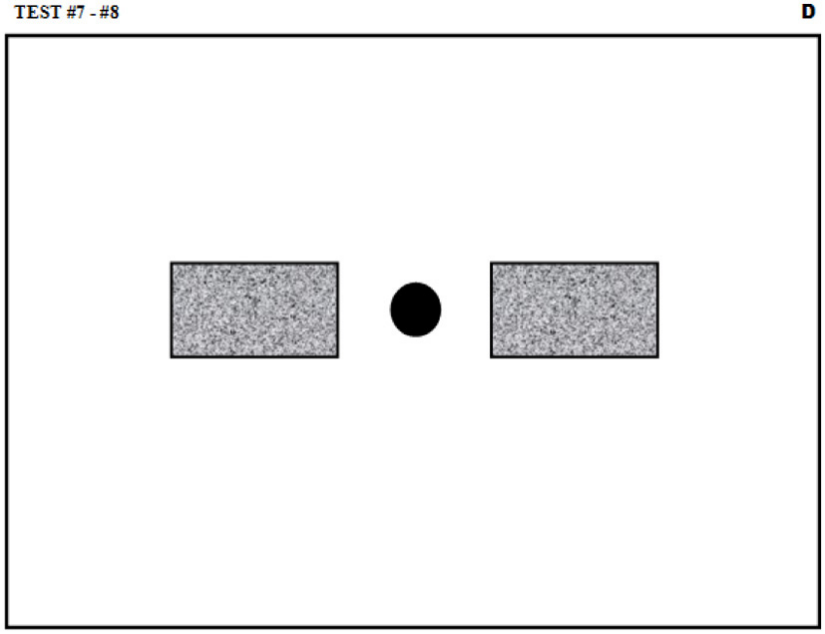
**Guiding sheet for the test involving oscillatory movements (#7 and #8)**. The black circle and the gray areas indicate respectively the starting and the target zones.

Oscillations are fundamental patterns in human behaviors. Accordingly, they have been well studied in the context of handwriting (Teulings and Maarse, 1984; Stelmach and Teulings, 1987). Movement models considering the motor control as composed of coupled oscillators has even been proposed (Yamanishi et al., 1980; Hollerbach, 1981; Gangadhar et al., 2007). Simple oscillatory movements have therefore been included in this battery with the hypothesis that neuromuscular degradation could be assessed by looking at some characteristics of such movements. More specifically, this test is performed to evaluate the rhythmical properties of the subject as his/her forearm or hand is oscillating at maximal frequency. Since only the speed is important in this test, large target zones have been defined in its guiding sheet (see Figure 7). No practice or learning period is allowed for this test to avoid generating muscular fatigue. For the same reason, only one acquisition is performed. After the subject is in position (in the starting area shown as a black circle), an auditory cue signals the start of the movement: he/she must oscillate the pen as fast as possible between the two gray zones. After 10 s, a long beep indicates the end of the trial.

#### Task #8: synchronized oscillations

This test evaluates the capacity of the subject to synchronize his/her movement with an auditory metronome. Its protocol is identical to the one of the task #7 except that after the start signal, the stimulator emits short beeps at a half-second interval for duration of 10 s. After this 10-s period, the auditory metronome stops but the subject must continue to move backward and forward the pen at the same frequency until the stop signal (a long beep) is emitted 5 s later. Two trials are recorded, the first one being a practice. The first part of the second trial is used to analyze the adaptation while the second part is used to study the steady state.

#### Task #9: final signatures

This final signature acquisition is performed as a mean to monitor if the neuromuscular state of the subject has been affected by the experiment. The protocol is identical as for test #1 except that only one signature is recorded. However, a second signature is collected if the Sign@matic software does not recognizes successfully the subject (e.g., the testing procedure has induced fatigue or influenced the motor control properties resulting in signatures at the end of the test that are significantly different than those produced at the onset of the experiment).

### Data collection

**Figure 8:**
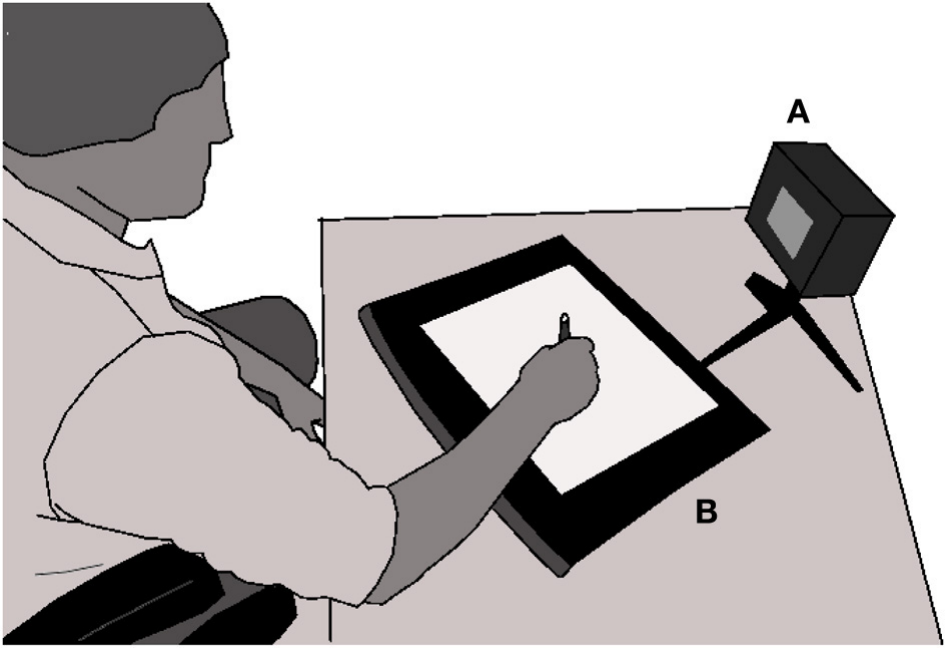
**Experimental setup showing the subject sited comfortably and ready to make a movement over the Wacom tablet (B) as soon as the stimulator (A) gives the cue**. Reproduced, with slight modifications, from O'Reilly et al. (2013).

For the data acquisition, the participant is invited to take a comfortable position and to place the tablet as wanted (see Figure 8). To normalize the stimulus perception, the stimulator is placed at a fixed distance of 68 cm from the border of the table and oriented such that its screen is perpendicular to the subject's line of sight.

The experimenter (not shown in Figure 8) is sitting at the right-hand side of the subject and demonstrates every task on its own set of guiding sheets. He also leads the subject as needed. Sited at the right of the experimenter and separated by an opaque panel to avoid distracting the subject, the system operator monitors the recording, verifies the validity of the recorded signals and informs the experimenter of any potential problem. He also has to record every event in a log book and discard manually invalid acquisitions that have not been detected automatically by the acquisition software. Frequent causes for rejecting movements are: (1) participant performing slow movements when fast movements are required; (2) participant not stopping his/her movement before lifting the pen from the tablet; (3) participant not lifting enough the pen from the tablet between two movements (for movement segmentation and experiment automation, the pen must exit the active zone of the Wacom digitizer between every trials); (4) participant starting his/her motion before the stimulus onset.

The total duration of the experiment is between 30 and 60 min, including subject welcoming and acknowledgement.

### Movement kinematics modeling

Collected movements have been modeled according to the Kinematic Theory of Rapid Human Movement (Plamondon, 1995a,b) used in numerous motor control studies in the last 20 years. This framework proposes that movements are controlled by a series of motor commands dispatched through the neuromuscular system, each command resulting in a lognormal contribution to the observed speed profile. As example, for the simplest movements analyzed here (i.e., the fast reaching motions associated with the reaction time tests), the resulting movement is considered as being the synergetic result of two neuromuscular systems. The first one, the agonist, pushes the end-effector (the pen tip) toward its target. The second one, the antagonist, has a directly opposed contribution and is mainly used to break the motion, although it can also be useful in stabilizing the movement and increasing its precision (Lestienne, 1979). Both neuromuscular systems are triggered at the same time *t*_0_ by commands of amplitude *D*_1_ and *D*_2_, respectively. These commands propagate through the neuromuscular systems and produce the lognormal impulse responses Λ_1_ and Λ_2_, respectively. With this modeling, the observed speed profile can be represented by the delta-lognormal curve which is expressed by

(1) $$\Delta \Lambda =D_{1}\Lambda_{1}-D_{2}\Lambda_{2}$$

with the Λ_*i*_ terms being time-shifted lognormals

(2) $$\Lambda_{i}=\frac{1}{\sigma_{i}\sqrt{2\pi }(t-t_{0})}exp\left(\frac{{\left(\ln\left(t-t_{0}\right)-\mu_{i}\right)}^{2}}{-2\sigma_{i}^{2}}\right)$$

The μ_*i*_ and σ_*i*_ parameters associated with the lognormal components represent the time delays and the response spreads (both on a logarithmic time scale) of the neuromuscular systems whereas the *t*_0_ and the *D*_*i*_ parameters are the command parameters.

The lognormal patterns of this model are naturally emerging from a converging behavior predicted by application of the central limit theorem, once applied to a system modeling of the neuromuscular networks. This representation considers a neuromuscular system as composed of a complex arrangement of subsystems which reaction to a motor command is cascaded such that they produce outputs with cumulative time delays following a law of proportionate effects (Plamondon, 1995a). This methodology has been validated, among other things, by the observation of an event-related potential (ERP) occurring at time *t*_0_ (O'Reilly et al., 2013) and by the experimental observation of a law of proportionate effects in EMG recordings (Plamondon et al., 2013a).

The model described in (1, 2) is appropriate for describing fast reaching movements and have therefore been used in analysing the kinematics of movements produced in task #2, #3, #4, and #5.

This modeling scheme can be generalized to model arbitrarily complex movements, such as handwritten signatures for example. To that end, a vector version of the previous representation is used. Such a data description is known as the Sigma-Lognormal modeling (Plamondon and Djioua, 2006; O'Reilly and Plamondon, 2009) and proposes a time superposition of lognormal neuromuscular components acting around circle-arc trajectories such that the velocity of the movement is described by

(3) $$\vec{v\left(t\right)}=\sum_{i=1}^{N}D_{i}\Lambda_{i}\left[\begin{array}{c} cos(\phi_{i}(t))\\ sin(\phi_{i}(t)) \end{array}\right]=\left[\begin{array}{c} v_{x}(t)\\ v_{y}(t) \end{array}\right]$$

with the movement direction being described using an error function (erf)

(4) $$\phi_{i}\left(t\right)=\theta_{si}+\frac{\left(\theta_{ei}-\theta_{si}\right)}{2}\left[1+erf\left(\frac{ln\left(t-t_{0i}\right)-\mu_{i}}{\sigma_{i}\sqrt{2}}\right)\right]$$

As shown in (4), this model introduces two supplementary parameters to described the trajectory of the movement in a plane, the starting (θ_*s*_) and the ending (θ_*e*_) trajectory direction angles. This model is adapted for the description of complex trajectories and has therefore been used to model the kinematics of movements gathered in task #1, #6, and #9.

As for the oscillatory motion recorded in task #7 and #8, they have been analyzed using a model which is half way between the Delta-Lognormal and the Sigma-Lognormal models: the Omega-Lognormal model. It analyzes the motion in one dimension as an alternate sequence of lognormals and is defined by

(5) $$\Omega \Lambda =\sum_{i=1}^{N}D_{1i}\Lambda \left(t-t_{01i};\mu_{1i},\sigma_{1i}\right)-\sum_{j=1}^{M}D_{2j}\Lambda \left(t-t_{02j};\mu_{2j},\sigma_{2j}\right)$$

with |*N* − *M*| ∈ {0, 1}.

### Parameter extraction

Parameter extractors are necessary to analyze movements using the lognormal models described in the preceding section. For the delta-lognormal parameters of task #2, #3, #4, and #5, the IIX system (Djioua and Plamondon, 2009) cascaded with a direct search optimization has been used. For the stereotypical patterns of the task #6, a prototype-based extractor (O'Reilly and Plamondon, 2010b) has been used to extract the sigma-lognormal parameters whereas the more polyvalent Xzero Robust extractor (O'Reilly and Plamondon, 2009) has been used for the signatures gathered in task #1 and #9. Finally, for oscillatory motions (task #7 and #8), a modified version of the Xzero Robust extractor has been used (O'Reilly, 2012).

### Statistical analysis

Various characteristics were defined to represent the neuromuscular health of our subjects. The most important are the central tendency (evaluated as the median) and the spread (evaluated as the median of the absolute deviation from the median) of the lognormal parameters (*t*_0_, *D*_*i*_, μ_*i*_, and σ_*i*_) and of the most frequently studied experimental measurements (e.g., reaction time, movement duration, amplitude and time occurrence of the maximum speed, etc.). The exact set of characteristics varied from a neuromuscular test to the other given the different characteristics of the motion associated with these different tests.

For each test, about 20 of these features were computed. Computation and evaluation of the usefulness of a large number of features has been avoided to reduce the probability of over-fitting. The best subset of a maximum of six features was defined using a semi-exhaustive selection algorithm (O'Reilly, 2012). A probability for the subjects to have the different brain stroke risk factors was evaluated using two techniques commonly used for medical diagnosis: the logistic regression and the *a posteriori* probability associated with the linear discriminant analysis (Tabachnick and Fidell, 2007). A leave-one-subject-out cross-validation was applied to get unbiased predictions from the logistic and the discriminant analysis. All statistical analyses were performed using the R statistical software.

To focus on the clinical usefulness of the developed tools in evaluating the brain stroke susceptibility rather than on the statistical significance of relationships between movement characteristics and risk factors, a pattern recognition approach has been used. The performances of the classifiers developed for this purpose have been assessed using the area under the receiver operating characteristic curve (AUC) instead of more classical techniques such as ANOVAs which are more appropriate when investigating statistical significance.

The reader interested in more details regarding this methodology can consult the previous technical publications on this topic (O'Reilly and Plamondon, 2011, 2012a,b; O'Reilly, 2012; Plamondon et al., 2014).

## Results

Table 1 reports the quality of risk factors classification based only on movement information. The results are measured using the AUC, a statistic corresponding to the probability of classifying correctly two subjects, knowing that one is having the risk factor and one is free of it. Scores of 0.5 and 1.0 are associated with a random and a perfect classification, respectively. A rule of thumb used in clinical setting makes a parallel with the traditional academic point system to qualify the quality of the AUC associated with a diagnostic test: 0.9–1.0 is excellent, 0.8–0.9 is good, 0.7–0.8 is fair, and 0.6–0.7 is poor (Tape, 2012). Although such a qualitative assessment depends on the problem at hand, this rule of thumb can be used as a starting point when no previous data are available to compare the AUC reached in a study with previously established results.

**Table 1:** **AUC for risk factors classification for the different neuromuscular tests**.

**Risk factor**	**Task #2**	**Task #3**	**Task #4**	**Task #5**	**Task #6**	**Task #7**	**Task #1&9**	**Median**
	**Visual SRT**	**CRT**	**Auditory SRT**	**SAT**	**Triangles**	**Oscillations**	**Signatures**	
DM	0.85	0.89	0.82	0.85	0.82	0.76	0.82	0.82
HT	0.76	0.76	0.76	0.74	0.80	0.77	0.76	0.76
HC	0.81	0.73	0.78	0.75	0.73	0.66	0.69	0.73
CS	0.69	0.72	0.82	0.71	0.70	0.60	0.34	0.70
CD	0.81	0.85	0.82	0.80	0.81	0.74	0.82	0.81
OB	0.78	0.85	0.88	0.73	0.68	0.75	0.73	0.75
Median	0.80	0.81	0.82	0.75	0.77	0.75	0.75	0.76

*Abbreviations: DM, diabetes mellitus; HT, hypertension; HC, hypercholesterolemia; CS, cigarette smoking; CD, cardiac disease; OB, obesity; SRT, simple reaction time; CRT, choice reaction time; SAT, speed-accuracy trade-off. Note: results for task #8 are not reported due to the fact that the analysis of this task was problematic because different strategies were used by different participants (e.g., fast movements followed by pauses vs. slower but continuous movements) and some subjects failed to oscillate at the requested frequency*.

In Table 1, the results for each neuromuscular test are shown in the different columns whereas the risk factors are associated with the rows. Median AUCs are given for each test (last row) and for each risk factor (last column) to help appreciate which tests are more discriminative and which risk factors are better discriminated. The cell at the lower right corner of the table gives the overall average.

AUC varying mostly between 0.6 and 0.9 have been obtained, the majority (83%) of these being over 0.7 and 35% being over 0.8. Looking at the Table 1 in more details, a few key observations must be pointed out. As can be seen in its rows, the best results are achieved for the discrimination of the diabetes and the cardiac disease with a mean AUC of 0.85 and 0.81, respectively. Hypertension, hypercholesterolemia, and obesity seem to be a little more difficult to assess using this methodology although the mean AUC of 0.77, 0.75, and 0.78 nevertheless indicates a significant relationship. Cigarette smoking is the least well characterized risk factor with a mean AUC of 0.61.

Results for task #2, #3, and #4 are bootstrapped averages and are all significantly larger than 0.5 (i.e., are better than chance) using a 95% confidence interval.

In looking at the columns of Table 1, we can see that some tests seem more discriminative than others. In general, better results have been obtained with simpler task (e.g., mean AUC between 0.78 and 0.81 for the SRT and CRT tests) than for complex ones (e.g., signatures with a mean AUC of 0.71).

**Figure 9:**
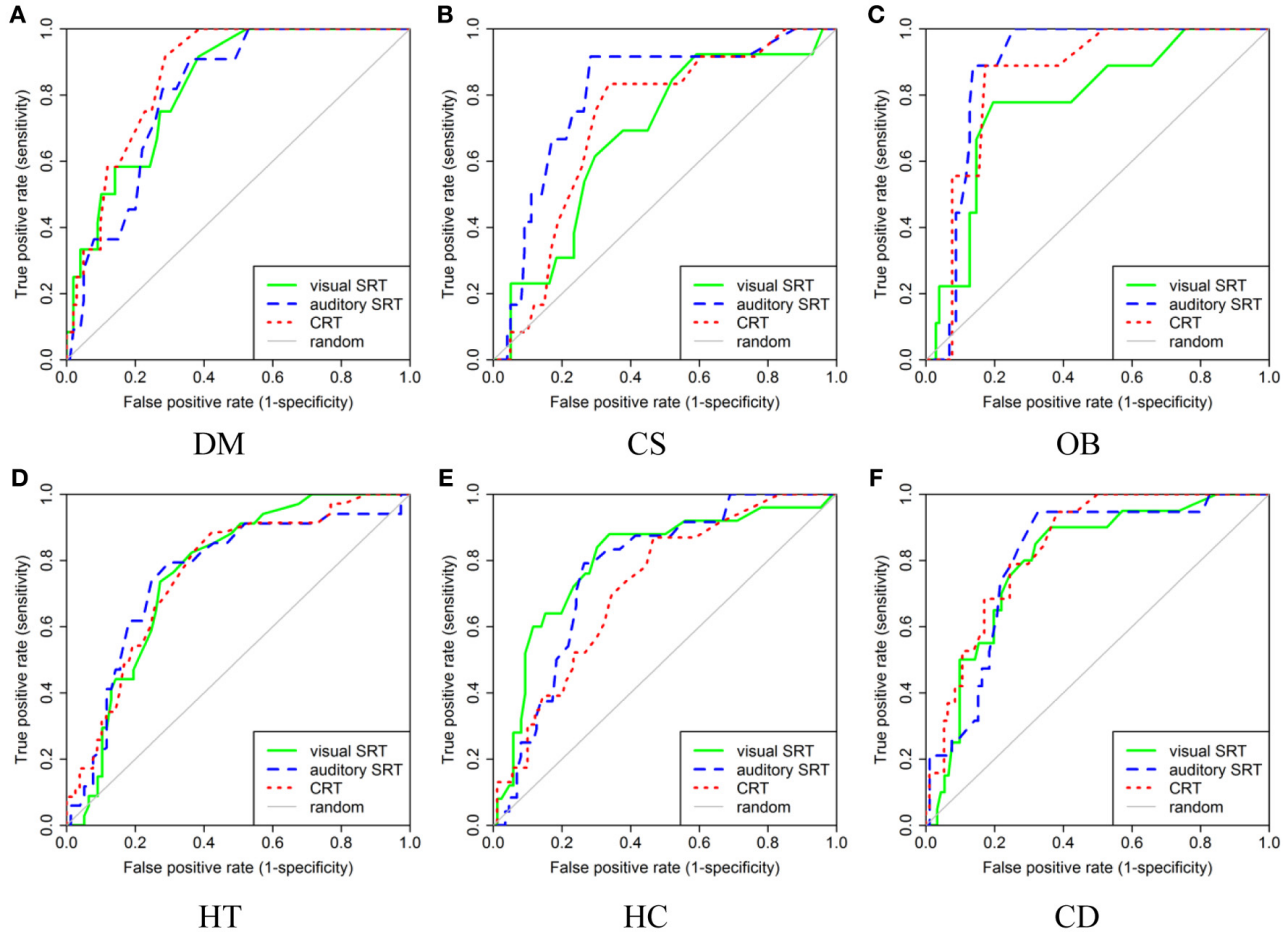
**ROC curve for the results obtained in visual SRT, auditory SRT, and CRT task for every risk factor: **(A)** DM, **(B)** CS, **(C)** OB, **(D)** HT, **(E)** HC, and **(F)** CD**.

AUCs are useful as they sum up the behavior of a classifier in one statistic which allows reporting a large number of results in little space, as in Table 1. They, however, are not as much flexible as the complete ROC curves. Although the 42 ROC curves associated with Table 1 AUCs could not be reported, the ROC curves associated with the task #2, #3, and #4 are presented in Figure 9 to allow the reader to better appreciate our results. Also, interested readers can consult the ROC curves related to task #6 in O'Reilly and Plamondon (2011). One should be aware, however, that the clinically usable portion of these curves is dependent on the risk factor prevalence in the targeted population and the minimal precision (or positive predictive value) that is acceptable (O'Reilly and Nielsen, 2013). For a given application, these two parameters should be set before choosing an operating point on these curves.

## Discussion

### Neuromuscular testing

In our investigation, simpler tasks were more predictive than complex ones. This might seem surprising since tasks requiring significantly more information processing were expected to better discriminate some risk factors, as shown in the case of nicotine abstinence (Marzilli and Shea, 2000). This difference of performance between simple and complex tasks might come from the larger number of repetitions collected for simple tasks (e.g., 15 for SRT and 30 for CRT) as compared to the complex ones (e.g., 5–7 for the signatures). Hence, we cannot conclude without any doubt on whether better results are to be attributed to the simplicity of the task, to a more robust modeling, or to the availability of more repetitions. However, comparable results have been found applying a similar methodology to assess the learning of kindergarten pupils (Duval et al., 2013; Duval et al., submitted).

Two supplementary comments are worth noting: First, regardless of the exact cause of the better discrimination obtained with simpler movements, the important variability of human movement encountered during our analyses suggests that it is preferable to collect large number of repetitions in order to achieve a good statistical characterization of the motor control. Therefore, future studies should consider many repetitions by experimental condition at the expense of reducing the number of different tasks. About 30 valid trials seem an adequate number when possible (i.e., for ethical reasons, it might be difficult to get that many repetitions from very young, very old, or severely diseased participants).

Second, given the length of the experiment (30–60 min), the fixed order of test presentation, and the fact that more complex movements are presented later in the experiment, the hypothesis that the test order could partially account for this higher discriminative power of simpler tests cannot be ruled out. However, results of unpublished work (e.g., our work with children) supporting the same observation with different tests presented in a different order suggest that at least part of this observation is not related to the test presentation order.

### Predictability of risk factors

It is worth noting that, on one hand, the consistency of the results across the different tests for some risk factors (e.g., the high AUC for the DM factor, the low AUC for the CS factor) supports the reliability of the AUC obtained for these risk factors. On the other hand, the variability of the results for some other factors (e.g., the AUC for the OB factor vary between 0.68 and 0.88, depending on the test) indicates one of two things: (1) that the tests are differentially assessing these risk factors or (2) that the observed variability is a statistical artifact (e.g., due to the relatively small number of subjects being affected by some of the analyzed risk factors). Our experiment will have to be duplicated to validate the reproducibility of these patterns before a decisive conclusion can be reached on that matter.

Our analyses also revealed a lower discrimination of cigarette smoking as compared to the other investigated risk factors. This might be the result of the opposing effects of long-term cigarette smoking and of short-term presence of nicotine in the blood stream. Whereas the first one has detrimental effects on motor control (Hill, 1989; Kalmijn et al., 2002), the second one has positive effects such as reduced reaction time (Davranche and Audiffren, 2002; Hahn et al., 2009) and increased vigilance (Ernst et al., 2001; Griesar et al., 2002). As neither the duration between the last cigarette consumption and the experiment nor the history of cigarette smoking have been controlled or registered in this experiment, it is not possible to discriminate between these two separate and opposite effects using a *post-hoc* analysis. This remark about short vs. long-term effects can be widened to the other risk factors highlighting the importance, in the planning of future experiments, to discriminate between the long and short term effects by controlling or recording the appropriate variables.

### Comments on the general approach

In this study, the presence of brain stroke risk factor is used as a proxy for brain stroke susceptibility as there is no gold standard index for stroke susceptibility. Although we present the diagnostic results for risk factors, this is not the end goal of this research program since many of these risk factors can easily be evaluated with high precision and reliability. Evaluating the possibility of diagnosing risk factors using movement characteristics is only used as a mean to show the potential of human movement characteristics for the evaluation of stroke susceptibility. The results reported herein are a first step necessary to build a sufficiently solid ground for including this type of assessment in longitudinal studies.

Such a prospective study will be important to improve further upon the figures of Table 1. For example, an important problem that needs to be addressed is the lack of a gold standard index of stroke susceptibility to compare the results of such new methodologies to (e.g., an index similar to the one proposed by Wang et al., 2003 for predicting heart stroke). The development of such an index is critical for the development of preventive tools and can only be evaluated soundly in such longitudinal investigations.

Considering the statistical interpretation of the AUC, our results support that there is a significant amount of information in the characteristics of human movements that is correlated with the brain stroke susceptibility. Of course, as of now, not all the information about the brain stroke risk factors can be extracted from motion analysis. As these results are obtained for a new trend of research, there is probably room for improvement but our present results are indicative of the potential of this technique. This methodology will need further fine tuning to lower the experimental error, to increase the statistical power of the analysis, and to augment the reliability of the results. Hopefully, as this approach is enhanced, the initial results published herein will improve and we will be in good position to rule on the final clinical usefulness of the movement analysis for the development of brain stroke prevention tools.

### Performance evaluation: pattern classification vs. analyses of variance

In this study, we have privileged using the pattern recognition techniques with AUC assessment rather than more classical analyses of variance (ANOVAs). This choice has been made because the classification approach does not aim *per se* at deciding whether or not a risk factor is related to the brain stroke susceptibility independently of any other confounding factors, it aims at determining how well we can predict stroke susceptibility for a representative set of subjects regardless of the presence of other correlated factors. In this context, the pattern recognition approach has the definitive advantage of being more indicative of clinical usefulness as it evaluates directly the diagnostic accuracy. It could be argued that a limitation of this approach is that it does not provide a framework which can easily control for the effect of external factors (e.g., age, gender)^3^. However, from the point of view of a classification problem, such a control of external effects has little sense and traditional analyses of variance can find statistically significant relationships that have no clinical usefulness because the effect size of the relationship is so weak that it cannot be used for any diagnostic application.

### Limitations

This experiment has some limitations that need to be addressed by future works. First, some potentially confounding variables were not recorded (e.g., the education level of the participants), which could be partly responsible for the association we observed between movement features and brain stroke risk factors. Regarding more specifically the education level of the participants, it must be noticed that the simplicity of the tasks and its easy acceptance and understanding by all the participants-particularly the aged ones who often refer to the whole protocol as a funny primary school tests-partly diminish the impact of this factor.

Second, this dataset is unbalanced in many regards, notably with respect to risk factor prevalence. The evidences gathered in this study about the relationship between stroke risk factors and the human movement characteristics supports the allocation of a more substantial financial support, a requirement for planning more extensive studies designed to gather larger and better balanced sample.

Third, for reasons discussed in previous subsections, we chose a pattern classification approach for this study. Further investigation of this dataset with statistical analysis of variance would nevertheless be very useful to complete and corroborate these results. Indeed, control and assessment of the impact of some confounding factors (e.g., age, gender, level of incapacity, education) is much easier in this framework than in the approach used in this paper. An effort to perform such an evaluation has been previously made in O'Reilly and Plamondon (2011) which describes, on the same data set and for the triangular drawing test, the impact of age and the gender on the relationship between movement features and brain stoke risk factors.

### Potential for future applications

The proposed computerized measurements may have a great potential as it could be implemented easily on tablets (e.g., iPad), intelligent phone, and so on. Thus, this technology might have a bright future not only for the neurologist, medical doctor or psychologist office, but also as a useful tool for telemedicine and personal health monitoring of aging people.

Moreover, this methodology can be used in context of other situations than brain stroke. For example, it is presently investigated for the development of preventive approaches in Parkinson disease (Van Gemmert et al., 2013) and Alzheimer disease (Impedovo et al., 2013) and it has been shown to be sensitive to aging in general (Plamondon et al., 2013b).

## Conclusion

This paper presents a brand new model-based approach using movement analysis for preventive medicine. It provides evidences that there are links between brain stroke risk factors and the characteristics of human movements when these are modeled using lognormal patterns. It is expected that these results will motivate researchers to further explore these new fields of investigation to establish an appropriate methodology. As the stroke susceptibility assessment based on human movement analysis is a novel area of research, efforts in defining an adequate methodological framework and improving it are critical. It is hoped that this paper will contribute to the establishment of such a framework. This fundamental work is motivated by the encouraging results that have been obtained, outcomes suggesting that the characteristics of human movements can be used as biomarkers for assessing the brain stroke susceptibility. In the long run, it is hoped that this research trend will allow the inclusion of movement bio-signals within new brain stroke prevention tools.

It should be emphasized that the objectives of our research program in general and of this study in particular is not in itself the diagnostic of brain stroke risk factors as there is already efficient tests for this purpose. It has to be seen in the wider perspective of studying the relationships that the brain stroke susceptibility and that a possible pre-stroke state both share with the characteristics of the motor control, keeping in mind the long-term goal of developing better ways to assess the risk of suffering from a stroke. This first investigation is an important step toward an eventual prospective study incorporating the tools we propose herein. In a final application, the movement information might be collected through the evermore ubiquitous movement tracking devices (computer mouse, camera, handheld devices) and integrated with other information such as the doctor's knowledge of the patient risk factors.

Further investigation will be necessary to better define the potential and the limitations of the proposed approach. These will have to corroborate the results reported herein and examine the properties of this measurement methodology, properties such as validity, specificity, and test-retest reliability. More attention will also have to be paid to (1) the pathophysiology linking the motor control to the brain stroke susceptibility, (2) the short vs. long-term effects of the risk factor on the movements, and (3) the possible complementariness vs. redundancy of the different neuromuscular tests used in our battery. Nevertheless, the results obtained so far are promising and will hopefully bring more attention to this innovative topic.

### Conflict of interest statement

The authors declare that the research was conducted in the absence of any commercial or financial relationships that could be construed as a potential conflict of interest.

## Footnotes

^1^It corresponds to the space where the tablet can still track the stylus although the pen tip is not in contact with the tablet anymore. It is about 2.5 cm above the surface in Wacom tablets.

^2^See Plamondon and Alimi (1997) for an exhaustive review of the research on this topic up to 1997.

^3^However, O'Reilly and Plamondon (2011) evaluated whether human movements contains information on the presence of stroke susceptibility that cannot be accounted by age and gender.

## References

[B1] Bohan M. J. Longstaff M. G. Van Gemmert A. W. A. Rand M. K. Stelmach G. E. (2003). Effects of target height and width on 2D pointing movement duration and kinematics. Motor Control 7, 278–289 12893958 10.1123/mcj.7.3.278

[B2] Bokura H. Kobayashi S. Yamaguchi S. Iijima K. Nagai A. Toyoda G. (2006). Silent brain infarction and subcortical white matter lesions increase the risk of stroke and mortality: a prospective cohort study. J. Stroke Cerebrovasc. Dis. 15, 57–63 10.1016/j.jstrokecerebrovasdis.2005.11.001 17904049

[B3] Caligiuri M. P. Mohammed L. (2012). The Neuroscience of Handwriting: Applications for Forensic Document Examination. Boca Raton, FL: Taylor and Francis 10.1201/b11703

[B4] Caligiuri M. P. Teulings H. L. Dean C. E. Niculescu A. B. Lohr J. (2009). Handwriting movement analyses for monitoring drug-induced motor side effects in schizophrenia patients treated with risperidone. Hum. Mov. Sci. 28, 633–642 10.1016/j.humov.2009.07.007 19692133 PMC2749075

[B5] Caligiuri M. P. Teulings H. L. Dean C. E. Niculescu A. B. 3rd. Lohr J. B. (2010). Handwriting movement kinematics for quantifying extrapyramidal side effects in patients treated with atypical antipsychotics. Psychiatry Res. 177, 77–83 10.1016/j.psychres.2009.07.005 20381875 PMC2859992

[B6] Chiu T.-T. Lin C.-L. Young K.-Y. Lin C.-T. Hsu S.-H. Yang B.-S. (2011). A study of Fitts' law on goal-directed aiming task with moving targets. Percept. Mot. Skills 113, 339–352 10.2466/05.06.25.PMS.113.4.339-352 21987931

[B7] Davranche K. Audiffren M. (2002). Effects of a low dose of transdermal nicotine on information processing. Nicotine Tob. Res. 4, 275–285 10.1080/14622200210141635 12215236

[B8] Djioua M. Plamondon R. (2009). A new algorithm and system for the characterization of handwriting strokes with delta-lognormal parameters. IEEE Trans. Pattern Anal. Mach. Intell. 31, 2060–2072 10.1109/TPAMI.2008.264 19762931

[B9] Duval T. Rémi C. Plamondon R. O'Reilly C. (2013). On the use of the sigma-lognormal model to study children handwriting, in Paper Presented at the 16th International Graphonomics Society Conference (Nara).

[B10] Ernst M. Heishman S. J. Spurgeon L. London E. D. (2001). Smoking history and nicotine effects on cognitive performance. Neuropsychopharmacology 25, 313–319 10.1016/S0893-133X(01)00257-3 11522460

[B11] Fairhurst M. C. Linnell T. Glenat S. Guest R. M. Heutte L. Paquet T. (2008). Developing a generic approach to online automated analysis of writing and drawing tests in clinical patient profiling. Behav. Res. Methods 40, 290–303 10.3758/BRM.40.1.290 18411552

[B12] Fitts P. M. (1954). The information capacity of the human motor system in controlling the amplitude of movement. J. Exp. Psychol. 47, 381–391 10.1037/h0055392 13174710

[B13] Fitts P. M. Peterson J. R. (1964). Information capacity of discrete motor responses. J. Exp. Psychol. 67, 103–112 10.1037/h0045689 14114905

[B14] Fondation des maladies du coeur. (2012). Statistiques - Fondation Des Maladies du Coeur du Canada. Available online at: http://www.heartandstroke.com/site/c.ikIQLcMWJtE/b.3483991/k.34A8/Statistics.htm (Accessed on Février 2, 2012).

[B15] Gangadhar G. Joseph D. Chakravarthy V. S. (2007). An oscillatory neuromotor model of handwriting generation. Int. J. Doc. Anal. Recognit. 10, 69–84 10.1007/s10032-007-0046-0

[B16] Griesar W. S. Zajdel D. P. Oken B. S. (2002). Nicotine effects on alertness and spatial attention in non-smokers. Nicotine Tob. Res. 4, 185–194 10.1080/14622200210123617 12028851

[B17] Hahn B. Ross T. J. Wolkenberg F. A. Shakleya D. M. Huestis M. A. Stein E. A. (2009). Performance effects of nicotine during selective attention, divided attention, and simple stimulus detection: an fMRI study. Cereb. Cortex 19, 1990–2000 10.1093/cercor/bhn226 19073624 PMC2733682

[B18] Hankey G. J. (1996). Impact of treatment of people with transient ischaemic attacks on stroke incidence and public health. Cerebrovasc. Dis. 6, 26–33 10.1159/000108068

[B19] Hill R. D. (1989). Residual effects of cigarette smoking on cognitive performance in normal aging. Psychol. Aging 4, 251–254 10.1037/0882-7974.4.2.251 2789756

[B20] Hollerbach J. M. (1981). An oscillation theory of handwriting. Biol. Cybern. 39, 139–156 10.1007/BF00336740

[B20a] Impedovo D. Pirlo G. Mangini F. M. Barbuzzi D. Rollo A. Balestrucci A. (2013). Writing generation model for health care neuromuscular system investigation, in Proceedings of the Tenth International Meeting on Computational Intelligence Methods for Bioinformatics and Bioststistics (CIBB 2013) (Nice: Nice Sophia Antipolis University).

[B21] Jax S. A. Rosenbaum D. A. Vaughan J. (2007). Extending Fitts' Law to manual obstacle avoidance. Exp. Brain Res. 180, 775–779 10.1007/s00221-007-0996-y 17562027

[B22] Kalmijn S. van Boxtel M. P. Verschuren M. W. Jolles J. Launer L. J. (2002). Cigarette smoking and alcohol consumption in relation to cognitive performance in middle age. Am. J. Epidemiol. 156, 936–944 10.1093/aje/kwf135 12419766

[B23] Kelly-Hayes M. Beiser A. Kase C. S. Scaramucci A. D'Agostino R. B. Wolf P. A. (2003). The influence of gender and age on disability following ischemic stroke: the Framingham study. J. Stroke Cerebrovasc. Dis. 12, 119–126 10.1016/S1052-3057(03)00042-9 17903915

[B24] Klimkowicz A. Dziedzic T. Polczyk R. Pera J. Slowik A. Szczudlik A. (2004). Factors associated with pre-stroke dementia: the cracow stroke database. J. Neurol. 251, 599–603 10.1007/s00415-004-0384-5 15164195

[B25] Kobayashi S. Okada K. Koide H. Bokura H. Yamaguchi S. (1997). Subcortical silent brain infarction as a risk factor for clinical stroke. Stroke 28, 1932–1939 10.1161/01.STR.28.10.1932 9341698

[B26] Lestienne F. (1979). Effects of inertial load and velocity on the braking process of voluntary limb movements. Exp. Brain Res. 35, 407–418 10.1007/BF00236760 456449

[B27] Levin M. F. (1996). Interjoint coordination during pointing movements is disrupted in spastic hemiparesis. Brain 119(Pt 1), 281–293 10.1093/brain/119.1.281 8624689

[B28] Lloyd-Jones D. Adams R. Carnethon M. De Simone G. Ferguson T. B. Flegal K. (2009). Heart disease and stroke statistics–2009 update: a report from the american heart association statistics committee and stroke statistics subcommittee. Circulation 119, e21–e181 10.1161/circulationaha.108.191261 19075105

[B29] Luce R. D. (1986). Response times - Their Role in Inferring Elementary Mental Organization, Vol. 1 New York, NY: Oxford Science Publications

[B30] Lum P. S. Burgar C. G. Kenney D. E. Van der Loos H. F. (1999). Quantification of force abnormalities during passive and active-assisted upper-limb reaching movements in post-stroke hemiparesis. IEEE Trans. Biomed. Eng. 46, 652–662 10.1109/10.764942 10356872

[B31] Marzilli T. S. Shea J. B. (2000). Effects of smoking abstinence on movement regulation. Percept. Mot. Skills 90, 624–630 10.2466/pms.2000.90.2.624 10833763

[B32] Mendoza J. Hansen S. Glazebrook C. M. Keetch K. M. Elliott D. (2005). Visual illusions affect both movement planning and on-line control: a multiple cue position on bias and goal-directed action. Hum. Mov. Sci. 24, 760–773 10.1016/j.humov.2005.09.002 16223538

[B33] Minegishi H. Takahashi M. (2001). Basic study on trajectories of reaching movements in children with learning disabilities, in Proceedings of the 23rd Annual International Conference of the Ieee Engineering in Medicine and Biology Society, Vol. 1–4, 23 (Istanbul), 1186–1189

[B34] O'Reilly C. (2012). Développement D'outils D'analyse de la Motricité Fine Pour L'investigation de Troubles Neuromusculaires: Théorie, Prototype et Mise en Application Dans le Contexte des Accidents Vasculaires Cérébraux. Ph.D. École Polytechnique, Montréal, QC

[B35] O'Reilly C. Nielsen T. (2013). Revisiting the ROC curve for diagnostic applications with an unbalanced class distribution, in Paper Presented at the The 9th International Workshop on Systems, Signal Processing and their Applications, Mazafran

[B36] O'Reilly C. Plamondon R. (2009). Development of a Sigma-Lognormal representation for on-line signatures. Pattern Recognit. 42, 3324–3337 10.1016/j.patcog.2008.10.017

[B37] O'Reilly C. Plamondon R. (2010a). A lognormal framework for human movement rehabilitation, in Rehabilitation Engineering, ed Tan Y. K. (Vukovar: IN-TECH), 157–172

[B38] O'Reilly C. Plamondon R. (2010b). Prototype-based methodology for the statistical analysis of local features in stereotypical handwriting tasks, in Paper Presented at the International Conference on Pattern Recognition (Istanbul).

[B39] O'Reilly C. Plamondon R. (2011). Impact of the principal stroke risk factors on human movements. Hum. Mov. Sci. 30, 792–806 10.1016/j.humov.2010.07.010 20888057

[B40] O'Reilly C. Plamondon R. (2012a). Design of a neuromuscular disorders diagnostic system using human movement analysis, in Paper presented at the The 11th International Conference on Information Sciences, Signal Processing and their Applications (Montreal, QC).

[B41] O'Reilly C. Plamondon R. (2012b). Looking for the brain stroke signature, in Paper presented at the 21st International Conference on Pattern Recognition (Tsukuba Science City).

[B42] O'Reilly C. Plamondon R. Landou M. K. Stemmer B. (2013). Using kinematic analysis of movement to predict the time occurrence of a evoked potential associated to a motor command. Eur. J. Neurosci. 37, 173–180 10.1111/ejn.12039 23331497

[B43] Plamondon R. (1994). The design of an on-line signature verification system: from theory to practice. Int. J. Pattern Recognit. Artif. Intell. Spec. Issue Signat. Verif. 8, 795–811

[B44] Plamondon R. (1995a). A kinematic theory of rapid human movements. Part I. Movement representation and generation. Biol. Cybern. 72, 295–307 7748959 10.1007/BF00202785

[B45] Plamondon R. (1995b). A kinematic theory of rapid human movements. Part II. Movement time and control. Biol. Cybern. 72, 309–320 7748960 10.1007/BF00202786

[B46] Plamondon R. Alimi A. M. (1997). Speed/accuracy trade-offs in target-directed movements. Behav. Brain Sci. 20, 279–303 10.1017/S0140525X97001441 10096999

[B47] Plamondon R. Djioua M. (2006). A multi-level representation paradigm for handwriting stroke generation. Hum. Mov. Sci. 25, 586–607 10.1016/j.humov.2006.07.004 17023083

[B48] Plamondon R. Djioua M. Mathieu P. A. (2013a). Time-dependence between upper arm muscles activity during rapid movements: observation of the proportional effects predicted by the kinematic theory. Hum. Mov. Sci. 32, 1026–1039 10.1016/j.humov.2012.07.006 23219167

[B49] Plamondon R. O'Reilly C. Ouellet-Plamondon C. (2014). Strokes against stroke—strokes for strides. Pattern Recognit. 47, 929–944 10.1016/j.patcog.2013.05.004

[B50] Plamondon R. O'Reilly C. Rémi C. Duval T. (2013b). The lognormal hand writer: improving, performing and declining. Front. Psychol. 4:945 10.3389/fpsyg.2013.00945 24391610 PMC3867641

[B51] Plamondon R. Yergeau P. Brault J.-J. (1992). A multi-level signature verification system, in Pixels to Features III, eds Impedovo S. Simon J. C. (Amsterdam; New York; London; Tokyo: Elsevier Publ. Co), 293–301

[B52] Prablanc C. Desmurget M. Grea H. (2003). Neural control of on-line guidance of hand reaching movements. Prog. Brain Res. 142, 155–170 10.1016/S0079-6123(03)42012-8 12693260

[B53] Schroter A. Mergl R. Burger K. Hampel H. Moller H. J. Hegerl U. (2003). Kinematic analysis of handwriting movements in patients with Alzheimer's disease, mild cognitive impairment, depression and healthy subjects. Dement. Geriatr. Cogn. Disord. 15, 132–142 10.1159/000068484 12584428

[B54] Simner M. L. Leedham C. G. Thomassen A. J. W. M. (1996). Handwriting and Drawing Research: Basic and Applied Issues. Amsterdam; Washington; Tokyo: IOS Press, Ohmsha

[B55] Stelmach G. E. Teulings H. L. (1987). Temporal and spatial characteristics in repetitive movement. Int. J. Neurosci. 35, 51–58 10.3109/00207458708987109 3623819

[B56] Tabachnick B. G. Fidell L. S. (2007). Using Multivariate Statistics, 5th Edn Boston; Montréal: Pearson: Allyn and Bacon

[B57] Tape T. G. (2012). Interprating Diagnostic Tests. Available online at: http://gim.unmc.edu/dxtests/Default.htm (Accessed 26 June, 2012).

[B58] Teulings H. L. Maarse F. J. (1984). Digital recording and processing of handwriting movements. Hum. Mov. Sci. 3, 193–217 10.1016/0167-9457(84)90011-3

[B59] Van Donkelaar P. (1999). Pointing movements are affected by size-contrast illusions. Exp. Brain Res. 125, 517–520 10.1007/s002210050710 10323299

[B60] Van Gemmert A. W. Adler C. H. Stelmach G. E. (2003). Parkinson's disease patients undershoot target size in handwriting and similar tasks. J. Neurol. Neurosurg. Psychiatry 74, 1502–1508 10.1136/jnnp.74.11.1502 14617705 PMC1738235

[B61] Van Gemmert A. W. A. Plamondon R. O'Reilly C. (2013). Using the Sigma-lognormal model to investigate handwriting of individuals with Parkinson's disease, in Paper Presented at the 16th International Graphonomics Society Conference (Nara).

[B62] Vaughan J. Barany D. A. Sali A. W. Jax S. A. Rosenbaum D. A. (2010). Extending Fitts' Law to three-dimensional obstacle-avoidance movements: support for the posture-based motion planning model. Exp. Brain Res. 207, 133–138 10.1007/s00221-010-2431-z 20931178

[B63] Wagner J. M. Rhodes J. A. Patten C. (2008). Reproducibility and minimal detectable change of three-dimensional kinematic analysis of reaching tasks in people with hemiparesis after stroke. Phys. Ther. 88, 652–663 10.2522/ptj.20070255 18326055

[B64] Wang T. J. Massaro J. M. Levy D. Vasan R. S. Wolf P. A. D'Agostino R. B. (2003). A risk score for predicting stroke or death in individuals with new-onset atrial fibrillation in the community: the Framingham Heart Study. JAMA 290, 1049–1056 10.1001/jama.290.8.1049 12941677

[B65] Werner P. Rosenblum S. Bar-On G. Heinik J. Korczyn A. (2006). Handwriting process variables discriminating mild Alzheimer's disease and mild cognitive impairment. J. Gerontol. B Psychol. Sci. Soc. Sci. 61, P228–P236 10.1093/geronb/61.4.P228 16855035

[B66] Wu J. L. Yang J. J. Honda T. (2010). Fitts' law holds for pointing movements under conditions of restricted visual feedback. Hum. Mov. Sci. 29, 882–892 10.1016/j.humov.2010.03.009 20659774

[B67] Yamanishi J. Kawato M. Suzuki R. (1980). Two coupled oscillators as a model for the coordinated finger tapping by both hands. Biol. Cybern. 37, 219–225 10.1007/BF00337040 7448245

[B68] Yancosek K. E. Mullineaux D. R. (2011). Stability of handwriting performance following injury-induced hand-dominance transfer in adults: a pilot study. J. Rehabil. Res. Dev. 48, 59–68 10.1682/JRRD.2010.04.0074 21328163

